# PB.44: Audit of interval cancers from family history breast screening

**DOI:** 10.1186/bcr3544

**Published:** 2013-11-08

**Authors:** R Dunn, S Astley, M Bydder, DG Evans, A Howell, J Sergeant, AJ Maxwell

**Affiliations:** 1 University of Manchester, UK; 2 Nightingale Centre and Genesis Prevention Centre, University Hospital of South Manchester, UK

## Introduction

A total of 267 women screened by the family history clinic (FHC) have been diagnosed with breast cancer since 1990. In 73 women the cancers were diagnosed in the interval between mammographic screens, which were performed every 1 to 2 years and single read. The aim of this audit was to compare performance with that of the NHS Breast Screening Programme (NHSBSP).

## Methods

Screening mammograms were found for 21 women, who had 22 interval cancers (one was bilateral). Thirteen had symptomatic mammograms available for comparison. The site of the cancer was known for eight of the remaining nine cases. Blinded review of the screening mammograms was performed and then comparison was made with the symptomatic mammograms or other available location data.

## Results

The screening mammograms were classified as follows: Category 0 (no symptomatic mammograms available), 9; Category 1 (screening mammograms normal), 7; Category 2A (subtle appearances, recall not justified), 2; Category 2B (subtle appearances, recall indicated), 2; and Category 3 (suspicious abnormality), 2. Of the eight Category 0 cases with a known tumour location, a subtle abnormality was visible in this area in one case but the remainder were normal. Thus the screening mammograms were normal in 14 of the 21 cases (67%) where the tumour location was known. Fifteen (68%) of the interval cancers were diagnosed within 12 months of the last mammogram.

## Conclusion

The proportion of Category 2 and 3 cases is similar to that seen in the NHSBSP. Double reading may reduce the number of these in the FHC population.

